# Length of stay and determinants of early discharge after facility-based childbirth in Cameroon: analysis of the 2018 Demographic and Health Survey

**DOI:** 10.1186/s12884-023-05847-4

**Published:** 2023-08-10

**Authors:** Jovanny Tsuala Fouogue, Aline Semaan, Tom Smekens, Louise-Tina Day, Veronique Filippi, Matsui Mitsuaki, Florent Ymele Fouelifack, Bruno Kenfack, Jeanne Hortence Fouedjio, Thérèse Delvaux, Lenka Beňová

**Affiliations:** 1 Faculty of Epidemiology and Population Health, London School of Hygiene & Tropical Medicine, London, UK; 2 Faculty of Medicine and Pharmaceutical Sciences, University of Dschang, Dschang, Cameroon; 3 Department of Public Health, Institute of Tropical Medicine, Antwerp, Belgium; 4 School of Tropical Medicine and Global Health, Nagasaki University, Nagasaki, Japan; 5 Department of Clinical Sciences, Higher Institute of Medical Technologies, Yaounde, Cameroon

**Keywords:** Hospital discharge, Length of stay, Postnatal care, Postpartum care, Maternal health, Newborn health, Childbirth, Caesarean section, Facility childbirth, Institutional childbirth

## Abstract

**Background:**

A minimum length of stay following facility birth is a prerequisite for women and newborns to receive the recommended monitoring and package of postnatal care. The first postnatal care guidelines in Cameroon were issued in 1998 but adherence to minimum length of stay has not been assessed thus far. The objective of this study was to estimate the average length of stay and identify determinants of early discharge after facility birth.

**Methods:**

We analyzed the Cameroon 2018 Demographic and Health Survey. We included 4,567 women who had a live birth in a heath facility between 2013 and 2018. We calculated their median length of stay in hours by mode of birth and the proportion discharged early (length of stay < 24 h after vaginal birth or < 5 days after caesarean section). We assessed the association between sociodemographic, context-related, facility-related, obstetric and need-related factors and early discharge using bivariate and multivariable logistic regression.

**Results:**

The median length of stay (inter quartile range) was 36 (9–84) hours after vaginal birth (*n* = 4,290) and 252 (132–300) hours after caesarean section (*n* = 277). We found that 28.8% of all women who gave birth in health facilities were discharged too early (29.7% of women with vaginal birth and 15.1% after a caesarean section). Factors which significantly predicted early discharge in multivariable regression were: maternal age < 20 years (compared to 20–29 years, aOR: 1.44; 95%CI 1.13–1.82), unemployment (aOR: 0.78; 95%CI: 0.63–0.96), non-Christian religions (aOR: 1.65; 95CI: 1.21–2.24), and region of residence—Northern zone aOR:9.95 (95%CI:6.53–15.17) and Forest zone aOR:2.51 (95%CI:1.79–3.53) compared to the country’s capital cities (Douala or Yaounde). None of the obstetric characteristics was associated with early discharge.

**Conclusions:**

More than 1 in 4 women who gave birth in facilities in Cameroon were discharged too early; this mostly affected women following vaginal birth. The reasons leading to lack of adherence to postnatal care guidelines should be better understood and addressed to reduce preventable complications and provide better support to women and newborns during this critical period.

## Background

Maternal mortality rate (MMR) in sub-Saharan Africa (SSA) is the highest worldwide despite substantial decline over the past three decades. Most countries in SSA are not on track to meet the Sustainable Development Goal (SDG) 3 target on MMR reduction [1, 2]. Historically, most programs to reduce MMR in SSA targeted antenatal and intrapartum periods, with positive results through increased antenatal care (ANC) coverage and proportion of facility-based childbirths [1, 3–5]. Nowadays, postpartum maternal deaths accounts for 55–65% of all maternal deaths partly because of the drop in antepartum and intrapartum mortality, and the risk of death remains high several months after childbirth [1, 6]. Therefore, inadequate maternal care during the postnatal period could undermine the hard-won gains in MMR from improved coverage and quality of ANC and skilled birth attendance.

The postnatal period is critical from the biological, health system and socio-cultural perspectives. Biologically, major recovery processes start immediately after childbirth and achieve restitution of the pre-pregnancy physiology by the end of the puerperium. Any deviation from the normal pathway to a healthy recovery can lead to serious complications and even mortality. Examples include uterine atony leading to postpartum hemorrhage or (de novo) postpartum pre-eclampsia or hypertension [7, 8]. In addition, some intrapartum complications can be missed during pregnancy and childbirth and diagnosed at advanced stages during early postnatal care (PNC) check-ups. Such complications are best illustrated by mental health challenges which can re-emerge up to one year following childbirth [9]. From a health system perspective, the postnatal period is the transition between pregnancy and the non-obstetric periods of women’s life in the continuum of care. It is recommended to counsel women on postpartum maternal and newborn care, and lifelong health topics like family planning, nutrition and prevention of communicable and non-communicable diseases [10, 11]. Moreover, focused care should start postnatally for pre-existing conditions and those that went undiagnosed during pregnancy, such as gestational diabetes. From a socio-cultural perspective, all communities have specific customs and norms to accommodate postpartum mothers and their newborns [12, 13].

Improving coverage and quality of PNC has become a global focus as a key lever to improve women’s and child’s health [10, 11]. The World Health Organization (WHO) issued recommendations for PNC in 1998, 2004, 2013, and 2022 [10, 11]. In the two latest editions, length of stay (LOS) of ≥ 24 h after uncomplicated facility-based vaginal birth was recommended and included among indicators of quality of maternal and newborn health services [10]. There is currently no WHO guidance on the minimum LOS following a caesarean section (CS). In high-income countries, women are typically discharged 2—4 days after uncomplicated CS and continuity of care is ensured through routine outpatient and/or home-based visits in the first days and weeks after discharge [14–16]. In SSA, while most women now give birth in health facilities (65%) coverage and quality of PNC remain suboptimal [17–20].

Cameroon, a lower-middle income country in SSA, has high maternal (427 maternal deaths per 100,000 live births) and morbidity rates [21–23]. Between 2004 and 2018, facility-based births rose from 59.0% to 67.0%, while population-level CS rate rose from 2.0% to 3.5% over the same period [23]. PNC for women has historically received less attention in health policies compared to other sexual, reproductive and maternal health topics, including family planning, comprehensive abortion care, ANC, emergency obstetric care, and prevention of mother-to-child transmission of the human immunodeficiency virus [21, 22]. The first national PNC guidelines were issued by the Ministry of Health in 2018 and remain in effect at the time of writing [17]. They recommend ≥ 24 h LOS after vaginal births but do not specify a minimum LOS following CS [17]. Cameroonian obstetricians agreed to not routinely discharge women before 5 days after uncomplicated CSs.

Early discharge from facilities following childbirth results from an interplay of individual, community and health system-related factors. Any intervention to improve the uptake of WHO and national recommendations on minimal LOS after childbirth should be informed by a thorough understanding of those factors. Little is known regarding the average LOS and determinants of early discharge in Cameroon [17–20]. The objectives of this study are to estimate the LOS following facility-based childbirth in Cameroon and to examine the determinants of early discharge.

## Methods

### Study design and data source

We conducted a cross-sectional study using Cameroon’s 2018 Demographic and Health Survey (DHS) data to examine the characteristics of live births during the 2013–2018 period. The 2018 DHS was a nationally representative population-based household survey. The sampling design followed a two-stage cluster strategy which we took into account in our statistical analysis. First, the country was divided into twelve survey domains: the ten administrative regions, the political capital (Yaounde) and the economic capital (Douala). Each survey domain except for Yaounde and Douala was subsequently divided into urban and rural strata. During the first sampling step, 470 clusters were systematically sampled in urban and rural strata using weighted probabilities proportionally to their number of households. In the second step, a simple random sampling of households in each selected cluster was undertaken. All consenting women of reproductive age (15—49 years) living in selected households or who spent the night preceding the survey in those households were interviewed.

### Study population

We included all women aged 15–49 years who had a live birth in the survey’s five-year recall period (2013–2018). For each woman, we retained her most recent live birth if it occurred in a health facility. Respondents with missing, unknown or implausible LOS (> 8 weeks) were excluded from the analysis.

### Conceptual framework

**Fig. 1:**
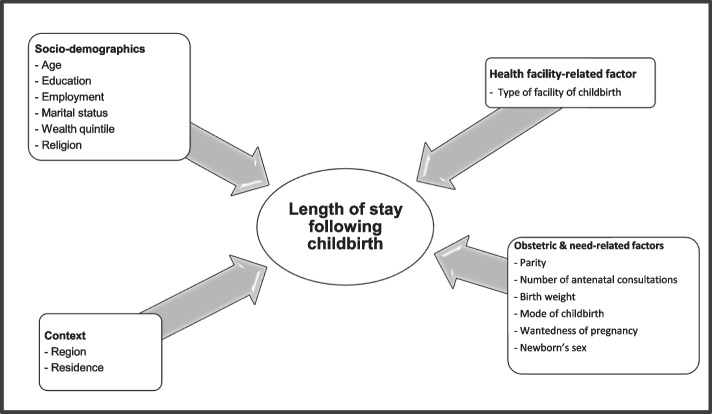
Conceptual framework of determinants of length of facility stay following facility childbirth

Building on results of a previous study, we hypothesized that LOS in health facility following childbirth is related to the interplay between several factors: contextual, socio-demographic, health facility, and obstetric and need-related [20]. Figure 1 illustrates the conceptual framework of determinants of LOS used in this study and shows the factors about which variables were available on the Cameroon 2018 DHS.

### Definitions

#### Outcome variables

During the DHS interview, LOS was captured by asking women: “How long after (name of the index child) was delivered did you stay in there (health facility)?”. Four possible answers were valid: “number of hours” (if less than one day), “number of days” (if at least one day and less than one week), “number of weeks” and “don’t know”. We used values expressed in hours as reported by women and transformed values in days and weeks into hours using the method described by Campbell et al. [20]. For example, LOS of one day was converted to 36 h (24 + 12), a stay of two days was converted to 60 h (48 + 12), and a stay of 3 days to 84 h (72 + 12). Similarly, LOS of one week was converted to 10.5 days (7 days + 3.5 days, which is 252 h). This transformation was done to account for the approximation based on the assumption that answers in days and weeks were, on average, longer in hours. We defined early discharge as a LOS < 24 h after vaginal birth and < 120 h (5 days) after CS.

#### Determinants

##### Socio-demographic factors

Age of the woman in years at the time of the index childbirth was computed and categorized in four groups (< 20, 20-29, 30-39, 40-49). The woman’s highest completed educational level was categorized as: no education, primary education, secondary education and higher education. Two categories were created to capture employment status of women at the time of the survey: employed (paid work) and unemployed. Marital status at time of survey was defined as married/cohabiting or not. The wealth quintile of the woman’s household was used as provided by the DHS dataset. Religion was categorized as Christianity (Catholic, Protestant or other Christian) or Islam/others (Animist, other and no religion).

##### Health facility-related factors

We re-coded facilities of childbirth into: 1. public hospitals (secondary and tertiary care health facilities), 2. public dispensaries (including integrated health centers and sub-divisional medical centers that are both primary care health facilities in Cameroon), 3. private health facilities (faith-based or for-profit facilities of any level of care), and 4. unknown (when the health facility type could not be ascertained by the respondent or survey enumerator). We did not include the qualification of the birth attendant for several reasons: anticipated collinearity with the type of health facility and mode of birth, poor criterion validity of recall, and the fact the birth attendant is not necessarily the provider involved in discharge [24].

##### Obstetric & need-related factors

Parity at the time of discharge was categorized as 1, 2–4, or 5 + . The number of ANC visits during the index pregnancy was categorized as 0 (no ANC), 1–4, and 4 + , according to national guidelines for focused ANC. Mode of birth was reported by the woman as either vaginal or CS. Newborn birthweight was assessed by the following questions: “Was (name of child) weighed at birth?” if yes, “How much did (name) weigh at birth?”. The answer was recorded in grams, either from memory alone or checked in the ANC booklet and categorized as < 2500 g, 2500–3999 g, or ≥ 4000 g. The sex of the newborn was male or female. Wantedness of the index pregnancy was captured by the question: “When you got pregnant, did you want to get pregnant at that time?”. We categorized the woman’s response as yes (wanted) or no (unwanted or mistimed).

##### Context-related factors

Regions provided in the DHS dataset were grouped into five larger socio-ecological zones: Northern (Adamawa, North and Far North), Forest (Centre, South and East), English-speaking (North-West and South-West), Capital cities (Yaounde and Douala), and West-littoral (West and Littoral regions). Type of residence was provided in the dataset (urban or rural).

### Statistical analyses

We performed statistical analyses using R version 4.2.0 (GNU's not Unix Generalized Public License version 2). The DHS design is a non-probability two-level stratified sampling. To ensure representativeness and accurate confidence intervals, the dataset provided a weight for each woman, which was used in analyses. We also adjusted for clustering and stratification. We described the characteristics of the study population; categorical variables were summarized using frequencies and percentages with 95% confidence intervals (95%CI). The number of observations with missing data was reported for each variable. We calculated the median LOS (and interquartile range) in hours and the percentage of women who were discharged early, stratified by mode of childbirth.

To evaluate the association between determinants and early discharge, we conducted bivariate and multivariate logistic regressions. We examined vaginal births and CS in the same model because of the small number of CS births. Variables associated with early discharge in binary analysis at *p* < 0.05 were included in the multivariable model. We obtained crude and adjusted odds ratios (OR) with 95%CI and accounted for the survey design.

## Results

### Characteristics of the study sample

**Fig. 2:**
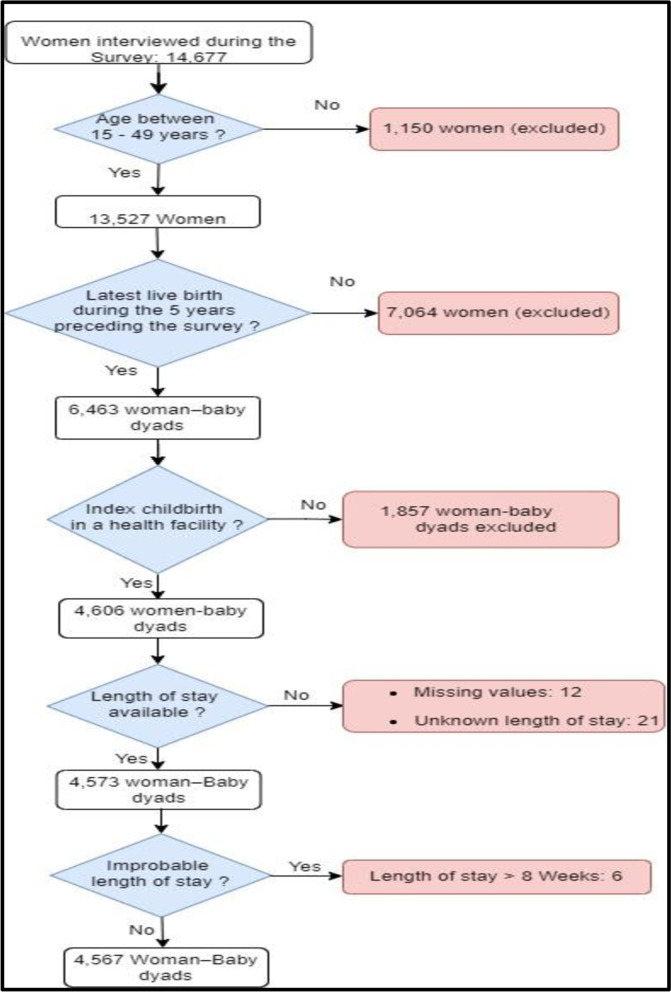
Study sample flow chart

Of the 14,677 women interviewed during the Cameroon 2018 DHS, 13,527 were aged 15–49 years and 6,463 had their latest live birth during the five years preceding the interview (Fig. 2). Home-based births accounted for 28.7% of the most recent live births (*n* = 1,857). Among the 4,606 facility-based births, LOS was missing for 12 women (four in public hospitals, three public dispensaries, and five in private health facilities) and was unknown by 21 women (four in public hospitals, fourteen in public dispensaries, and three in private health facilities). In addition, six participants (three in public hospitals, one in a public dispensary, and two in private health facilities) reported implausibly long LOS (> 8 weeks). These 39 women were excluded from the final analysis sample of 4,567 woman-baby dyads (Fig. 2).

Table 1 summarizes the characteristics of the study sample. Data were missing for two variables—number of antenatal consultations (*n* = 60, 1.3%) and birthweight (*n* = 300, 6.6%). Women in the study sample were predominantly aged between 15–30 years (67.9%), had some level of formal education (88.6%), were employed (72.3%), married/cohabiting (76.2%) and Christian (77.1%). Most were primiparous (60.8%) and reported receiving 4 + ANC consultations during the index pregnancy (80.0%). CS accounted for 6.1% of all births in this sample of women who gave birth in health facilities.

**Table 1:** Characteristics of study sample (*n* = 4,567)

Variables (number observations with missing data)	n (%)
**Socio-demographic characteristics**	
**Age group at index childbirth in years (0)**	
< 20	774 (15.7)
20 – 29	2363 (52.2)
30 – 39	1296 (29.3)
40 – 49	134 (2.8)
**Completed educational level (0)**	
No education	462 (11.4)
Primary education	1399 (30.0)
Secondary education	2340 (49.9)
Higher education	366 (8.7)
**Employment at time of survey (0)**	
Employed	3234 (72.3)
Unemployed	1333 (27.7)
**Marital status (0)**	
Married/cohabiting	3394 (76.2)
Not married/cohabiting	1173 (23.7)
**Household wealth quintile (0)**	
Q1 (Poorest)	328 (8.5)
Q2	837 (18.0)
Q3	1189 (22.4)
Q4	1201 (26.6)
Q5 (Richest)	1012 (24.5)
**Religion (0)**	
Christianity	3599 (77.1)
Islam	860 (20.6)
Animism	24 (0.6)
Other	84 (1.7)
**Health system factor**	
**Type of childbirth facility (0)**	
Public hospitals	1260 (26.7)
Public dispensaries	1808 (38.6)
Private health facilities	1499 (34.7)
**Obstetric and need-related factors**	
**Newborn’s sex (0)**	
Male	2341 (52.2)
Female	2226 (47.8)
**Parity (0)**	
1	2820 (60.8)
2 – 4	1744 (39.2)
≥ 5	3 (< 0.1)
**Multiplicity of the pregnancy (0)**	
Singleton	4437 (97.2)
Multiple	130 (2.8)
**Mode of Childbirth (0)**	
Vaginal	4290 (93.9)
Caesarean section	277 (6.1)
**Wantedness of pregnancy (0)**	
Yes	3381 (74.9)
No (mistimed or unwanted)	1186 (25.1)
**Number of antenatal care consultations (60)**	
0	102 (2.1)
1 – 3	877 (17.9)
≥ 4	3528 (80.0)
**Birthweight (300)**	
< 2500 g	265 (6.4)
2500 – 3999 g	3060 (71.5)
≥ 4000 g	942 (22.1)
**Contextual factors**	
**Region (0)**	
Adamawa	237 (3.4)
Centre (without Yaounde)	548 (12.0)
Douala	417 (13.4)
East	373 (5.8)
Far – North	313 (9.9)
Littoral (without Douala)	367 (4.8)
North	307 (7.9)
North-West	311 (8.2)
West	585 (14.0)
South	501 (5.6)
South-West	127 (2.2)
Yaounde	481 (12.8)
**Socio-ecological zone (0)**	
Northern zone (Adamawa, North, Far North)	857 (18.8)
Forest zone (Centre, South, East)	1422 (31.1)
Capital cities (Yaounde—Douala)	898 (19.7)
English speaking zone (North West and South West)	438 (9.6)
West-Littoral zone (West and Littoral)	952 (20.8)
**Residence (0)**	
Urban	2695 (61.7)
Rural	1872 (38.3)

### Length of stay following facility-based childbirth

Table 2 shows the median LOS in hours and the percentage of women discharged early. Among women who gave birth vaginally (*n* = 4,290), the median LOS was 36 h, while among those with a CS (*n* = 277), the median was 252 h (equivalent of 10.5 days). We estimated that 29.7% of women with vaginal births were discharged too early (< 24 h), as were 15.1% of women with a CS (< 120 h). Overall, we found that 28.8% of women who gave birth in health facilities were discharged early.

**Table 2:** Median length of stay and percentages of early discharge after facility-based childbirth in Cameroon, overall and by mode of birth (*n* = 4,567)

**Variables**	**Overall** *n* = 4,567	**Vaginal birth** *n* = 4,290	**Caesarean section** *n* = 277
Median length of stay in hours (inter quartile range)	60 (11 – 84)	36 (9—84)	252 (132—300)
Proportion of women with early discharge^a^ (95%CI)	28.8% (27.5—30.1)	29.7% (28.3—31.1)	15.1% (11.3—19.8)

^a^Cut-off for early discharge was: vaginal birth < 24 h and cesarean section < 120 h. 95%CI: 95% confidence interval

The cumulative distribution of LOS is shown in Table 3, disaggregated by mode of birth. Among women who had a vaginal birth, very early facility discharge (< 2 h) was reported by 9.7% of women, while 21.6% reported being discharged within 6 h. LOS of four or more days was reported by 7.5% of women. Among women who had a CS, 2.7% reported having been discharged on the day of childbirth. Those discharged within 3 days accounted for 7.2% and 26.2% were discharged longer than 7 days after birth.

**Table 3:** Distribution of length of stay after facility-based childbirth in Cameroon by mode of childbirth (*n* = 4,567)

**LOS in completed days**	**LOS in hours**	**Vaginal births** *n* = 4290		**Caesarean births** *n* = 277		**All births** *n* = 4567	
		**n (%)**	**Cumulative %**	**n (%)**	**Cumulative %**	**n (%)**	**Cumulative %**
0 (Day of birth)	0—2	393 (9.7)	9.7	2 (0.9)	0.9	395 (9.1)	9.1
	3—6	490 (11.9)	21.6	1 (0.7)	1.6	491 (11.2)	20.3
	7 – 12	255 (6.3)	27.9	1 (0.3)	0.3	256 (5.9)	26.2
	13 – 24	117 (2.7)	30.6	2 (0.8)	2.7	119 (2.6)	28.8
1	25 – 48	938 (19.8)	50.8	6 (1.2)	3.9	945 (18.7)	47.5
2	49 – 72	832 (18.9)	69.3	8 (3.3)	7.2	840 (17.9)	65.4
3	73 – 96	936 (23.2)	92.5	11 (4.1)	11.3	947 (22.1)	87.5
4	97 – 120	126 (2.9)	95.4	8 (3.8)	15.1	134 (3.0)	90.5
5	121—144	51 (1.0)	96.4	38 (14.5)	29.6	89 (1.8)	92.3
6	145 – 168	9 (0.2)	96.6	11 (4.3)	33.9	20 (0.5)	92.8
≥ 7	169—252	96 (2.1)	98.7	113 (39.9)	73.8	209 (4.4)	97.2
	≥ 253	47 (1.3)	100.0	76 (26.2)	100.0	123 (2.8)	100.0

*LOS* Length of stay

### Determinants of early discharge

Table 4 shows the crude and adjusted associations between determinants and early discharge for all births combined.

**Table 4:** Determinants of early discharge after facility-based childbirth in Cameroon, all modes of delivery included (*n* = 4,567)

**Factors**	**Crude analysis**		**Multivariable analysis**	
	**OR (95%CI)**	***p*-value**	**aOR (95%CI)**	***p*-value**
**Age group**				
< 20	1.63 (1.35—1.96)	< 0.001	1.44 (1.13—1.82)	0.003
20 – 29	Reference		Reference	
30 – 39	0.98 (0.82—1.17)	0.831	1.03 (0.82—1.29)	0.800
40 – 49	1.11 (0.73—1.68)	0.619	1.08 (0.70—1.66)	0.722
**Completed education level**				
No education	6.83 (4.89 – 9.55)	< 0.001	1.41 (0.99 – 2.00)	0.053
Primary	1.88 (1.56 – 2.26)	< 0.001	1.20 (0.99—1.46)	0.062
Secondary	Reference		Reference	
Higher	0.62 (0.43 – 0.90)	0.013	1.08 (0.71—1.63)	0.723
**Religion**				
Christianity	Reference		Reference	
Islam and others	3.40 (2.58—4.43)	< 0.001	1.65 (1.21—2.24)	0.001
**Marital status**				
Not married/cohabiting	0.66 (0.53—0.81)	< 0.001	1.04 (0.83—1.31)	0.712
Married/cohabiting	Reference		Reference	
**Household wealth quintile**				
Q1 (Poorest)	Reference		Reference	
Q2	0.31(0.20- 0.48)	< 0.001	0.84 (0.54—1.30)	0.426
Q3	0.23 (0.15—0.36)	< 0.001	0.76 (0.49 -1.19)	0.236
Q4	0.17 (0.11—0.28)	< 0.001	0.83 (0.50—1.38)	0.474
Q5 (Richest)	0.09 (0.06—0.15)	< 0.001	0.66 (0.39—1.12)	0.120
**Employment**				
Employed	Reference		Reference	
Unemployed	0.79 (0.65 – 0.96)	< 0.001	0.78 (0.63 – 0.96)	0.020
**Type of childbirth facility**				
Public hospital	0.35 (0.28—0.45)	< 0.001	0.65 (0.50—0.84)	0.001
Public dispensary	Reference		Reference	
Private health facility	0.41 (0.32—0.53)	< 0.001	0.81 (0.60—1.08)	0.153
**Newborn’s sex**				
Male	Reference		NA	NA
Female	0.90 (0.78—1.08)	0.232		
**Parity**				
1	0.74 (0.64—0.86)	< 0.001	0.94 (0.77- 1.14)	0.514
2 – 4	Reference		Reference	
≥ 5	1.86 (0.16—21.36)	0.616	3.99 (0.56 – 31.07)	0.186
**Mode of Childbirth**				
Vaginal	Reference		Reference	
Cesarean section	0.42 (0.28—0.63)	< 0.001	0.99 (0.56—1.47)	0.695
**Wantedness of pregnancy**				
Yes	Reference		NA	NA
No	0.84 (0.69—1.02)	0.072		
**Number of ANC visits**				
0—3	Reference		Reference	
≥ 4	0.49 (0.39—0.61)	< 0.001	0.89 (0.70—1.14)	0.349
**Birthweight**				
< 2500 g	0.94 (0.68—1.30)	0.704	NA	NA
2500 – 3999 g	Reference			
≥ 4000 g	1.14 (0.95—1.36)	0.149		
**Residence**				
Urban	Reference		Reference	
Rural	1.99 (1.54—2.57)	< 0.001	0.94 (0.70—1.26)	0.671
**Zone**				
Northern	19.3 (13.27—28.04)	< 0.001	9.95 (6.53—15.17)	< 0.001
Forest	3.27 (2.43—4.39)	< 0.001	2.51 (1.79—3.53)	< 0.001
Capital cities	Reference		Reference	
English speaking	0.49 (0.30—0.78)	0.003	0.38 (0.23—0.64)	< 0.001
West-Littoral	1.67 (1.04—2.69)	0.034	1.17 (0.78—1.75)	0.449

*NA* Not applicable. *Q* Quintile, *ANC* Antenatal care. *P* value of Wald test. *OR* Odds Ratio, *aOR* adjusted Odds Ratio (variables in the adjusted model: age, mode of birth, socioecological zone, Marital status, Religion, Parity, employment, type of childbirth facility, number of ANC visits, completed education level, household wealth quintile residence)

#### Bivariate analysis

Women who had their first child, gave birth by CS, were ≥ 20 years old and reported ≥ 4 ANC visits were significantly less likely to be discharged early. Compared to women who gave birth in public dispensaries, those who gave birth in private health facilities (OR:0.41; 95%CI: 0.32–0.53) and public hospitals (OR:0.35; 95%CI: 0.28–0.45) were less likely to be discharged early. Unemployed and unmarried women were less likely to be discharged early compared to employed and married women, respectively. Living in a rural area doubled the odds of early discharge compared to urban areas. Compared to the two capital cities, living in the Northern zone and in the Forest zone increased the risk of early discharge, while living in the English speaking zone reduced it. Wantedness of pregnancy, newborn’s sex and birthweight were not significantly associated with early discharge.

#### Multivariable analysis

In multivariable analysis, the following factors were no longer significantly associated with early discharge: marital status, household wealth quintile, parity, mode of birth, number of ANC visits, and residence. On the other hand, predictors which remained significantly associated with early discharge were age < 20 years (compared to 20–29 years: aOR: 1.44; 95%CI: 1.13–1.82), unemployment (aOR:0.78; 95%CI: 0.63 – 0.96), religion other than Christianity (aOR: 1.65; 95CI: 1.21–2.24), and geographic zone. Public hospitals remained protective against early discharge while private health facilities were no longer different from public dispensaries following adjustment. Primary education remained marginally associated with 20% higher odds of early discharge compared to secondary education.

## Discussion

We found that too many women left health facilities too early after childbirth in Cameroon. Nearly one in three women who gave birth in a health facility was discharged too early to benefit from evidence-based PNC and observation. This percentage was double among women with vaginal births compared to CS. Socio-demographic characteristics (adolescence, unemployment and non-Christian religions) and the type of health facility (public dispensary) were associated with higher odds of early discharge, while none of the obstetric characteristics was associated with early discharge. The zone of residence was also associated with early discharge.

### Length of stay following childbirth

The median LOS for vaginal births and for all women in our study were similar to findings by Campbell et al. in the following SSA countries: Benin, Ghana, Kenya, Liberia, Madagascar, Namibia, Nigeria, Sierra Leone, Uganda and Zambia [19]. Outside SSA, Kumar et al. reported similar LOS following both vaginal and caesarean births in India [25]. LOS following CS in Cameroon was much longer than in Europe and North America [14, 15, 26]; this is likely due to higher severity of complications among women who give birth by a CS, necessitating a longer period of in-patient care and observation [27]. There are several pathways for women to leave health facilities following childbirth: discharge by care providers (based on maternal and/or newborn clinical need and other factors, including payment of fees), referral to another facility (for maternal and/or newborn indication), and self-discharge. The prevalence of early discharge following vaginal births in our study was twice higher than following CS and the difference was significant in bivariate analysis. This can be explained by the difference in risks (perception by women and care providers) associated with CS births. On the users’ side, after uncomplicated vaginal births, women might be more prone to self-discharge against providers’ advice than those who had CS. On the supply side, providers may tend to delay discharge after CS to ensure that the least of medical criteria for safe discharge are met because of the absence of routine home-based PNC and low uptake of early outpatient PNC in Cameroon. In case of facility overcrowding, providers tend to prioritise discharge of women who had vaginal births.

In contrast to early discharge which was the main focus of this study, the case of women with LOS which seemed “too long” must be mentioned. In this study, 7.5% of women with a vaginal birth were discharged after five days or longer, and one in four women who had a CS left the hospital beyond the tenth postoperative day. Unnecessarily long LOS increases the risk of nosocomial infections, out-of-pocket costs (higher for CS), and disrupts early postnatal socio-cultural schemes at home, such as breastfeeding the newborn and care for other children [28]. Longer than expected LOS may be medically necessary in case of maternal or neonatal complications, but several non-medical factors might also be at play: detention for inability to pay fees, administrative red tape for discharge formalities, waiting time for the first newborn consultation by a pediatrician (for example, if not available during weekends), waiting time for newborn birth declaration, and waiting time for the first vaccine of the newborn which is often delayed by nationwide stock outs [25, 28, 29].

### Determinants of early discharge

Our results show that adolescents (< 20 years old) were more likely to depart the facility of birth early compared to older women. Campbell et al. reported similar results in several SSA countries while Kumar et al. found no relationship between maternal age and LOS in India [20, 30]. On the contrary, Pereira et al. and McMahon et al. reported that adolescence was significantly associated with longer LOS following CS in Brazil and Tanzania, respectively [31, 32]. Our findings were unexpected because the higher prevalence of childbirth complications among adolescents should necessitate longer LOS than in adults [33–35]. We suggest possible reasons include adolescent mothers leaving early due to stigma or to avoid additional expenses. Further studies are needed to provide a better understanding of this finding; in the meantime, clinicians should pay a particular attention when discharging adolescent mothers to ensure that the recommended minimal LOS and high-quality care are met. Reasons why Christian women were discharged in due time and whether/how other socio-economic factors are causing residual confounding in this association should also be examined in further studies.

Concerning contextual factors, early discharge was significantly more likely in Northern and Forest zones which have the highest percentages of home-based childbirth (ranging from 20.2% – 63.0%) and the lowest coverage of maternal health services in the country [36, 37]. We hypothesize that in those zones, the few women who give birth in health facilities might be more likely to advocate for earlier (self-)discharge in line with regional cultural expectations. Self-discharge might be one of the drivers of the much higher odds of early discharge in the remote and rural Northern zone, where women might live far from health facilities and rely on infrequent transport. In these poorest zones of the country, the infrastructure (space, beds, privacy, water and sanitation) in health facilities might be suboptimal and could have motivated early self-discharge by women, as it was documented in rural Tanzania [32]. Health authorities should focus on those geographic zones when implementing the current national guidelines on LOS and PNC.

In bivariate analysis, living in a rural area increased by two folds the probability of early discharge; this is similar to results reported by McMahon et al. in a rural region of Tanzania where 90% of women with uncomplicated vaginal births were discharged within 24 h [32]. However, the association between rural residence and early discharge lost significance after multivariable analysis like in the sub-Saharan African multi-country study by Campbell et al. [20]. Women who had vaginal birth in public dispensaries where significantly more likely to be discharged early than those in public hospitals. McMahon et al. reported similar results in Tanzania [32]. This could be explained by higher proportions of complicated births in hospitals which serve as referral facilities, warranting longer stays for adequate management compared to dispensaries, where most women have few or no complications. Other possible reasons include the higher qualifications of health care providers in hospitals resulting in better compliance to national guidelines, and use of lengthy and rigorous procedures for discharge. Dispensaries and other primary care facilities should be the priority target of any strategy to increase the uptake of recommendations on LOS following facility-based birth.

The drivers and root causes of the high prevalence of early discharge following facility-based childbirths in Cameroon have not been explored to date. To that end, we suggest further qualitative research like that carried out by McMahon et al. in Tanzania, which resulted in identification of drivers pertaining both to the supply and demand sides: overcrowding of health facilities, women’s physical and mental discomfort, self-discharge, pressing household commitments and routine practices (closing time and group discharge of low-risk women to accommodate others) [32]. Such study would provide insights into the elements of PNC which are not properly delivered when women are discharged early and help advocate for improvements.

### Strengths and limitations

This study is the first to assess LOS following facility birth in Cameroon and determinants of early discharge. It used a large nationally representative sample with high completion rates, which makes the results generalizable. Nevertheless, it has some limitations. Firstly, there is a possible recall bias because information was self-reported and collected retrospectively about an event dating up to five years earlier. The extent of validity and reliability of the LOS question has not been tested prior to the survey, women might not remember their exact LOS, and there was substantial heaping in the responses. In addition, the formula we used to transform LOS from days and weeks to hours is an approximation which has not been validated against direct prospective measurements. Secondly, social desirability bias could have impeded some answers because the interviewers were publicly announced and endorsed by public authorities. Women with an early (self-)discharge might have reported a longer length of stay, thereby artificially reducing the prevalence of early discharge. Thirdly, reliance on secondary data made it impossible to explore a broader range of factors associated with early discharge; especially those related to facility, health providers, women’s health status and content of care received by women during their postnatal stay in health facilities. Finally, the wording of the survey question to capture the LOS did not specify whether women stayed in the health facility for their own care or for the sick newborns.

## Conclusion

This first study to assess LOS in Cameroon found that more than a quarter of women who gave birth in health facilities were discharged too early as per the recommendations of the Ministry of Health, suggesting a misalignment between guidelines and practice. Several associated factors have been identified but root causes and drivers of early discharge and their concrete impact on completeness of recommended postnatal care content are still to be determined in view of informing response policies and strategies to improve the quality of postnatal care.

## Data Availability

The data that support the findings of this study are available from The Inner City Fund International Demographic and Health Survey Program (https://www.dhsprogram.com/data/dataset_admin/login_main.cfm) but restrictions apply to the availability of these data, which were used under license for the current study, and so are not publicly available. Data are however available from the corresponding author upon reasonable request and with permission of The Inner City Fund International Demographic and Health Survey Program.

## Abbreviations

ANC: Antenatal Care
aOR: Adjusted Odds Ratio
CS: Caesarean section
DHS: Demographic and Health Survey
LOS: Length of stay
MMR: Maternal mortality rate
NA: Not applicable
OR: Odds Ratio
PNC: Postnatal Care
SDG: Sustainable Development Goal
SSA: Sub-Saharan Africa
WHO: World Health Organization
